# Muscular and Tendon Degeneration after Achilles Rupture: New Insights into Future Repair Strategies

**DOI:** 10.3390/biomedicines10010019

**Published:** 2021-12-23

**Authors:** Lara Gil-Melgosa, Jorge Grasa, Ainhoa Urbiola, Rafael Llombart, Miguel Susaeta Ruiz, Verónica Montiel, Cristina Ederra, Begoña Calvo, Mikel Ariz, Purificación Ripalda-Cemborain, Felipe Prosper, Carlos Ortiz-de-Solórzano, Juan Pons-Villanueva, Ana Pérez Ruiz

**Affiliations:** 1 Orthopedic Surgery Department, Clínica Universidad de Navarra (CUN), 31008 Pamplona, Spain; lgil@unav.es (L.G.-M.); rllombartb@unav.es (R.L.); vmontiel@unav.es (V.M.); pripalda@unav.es (P.R.-C.); 2 Regenerative Medicine Program, Foundation for Applied Medical Research (FIMA), University of Navarra (UNAV), 31008 Pamplona, Spain; msusaetarui@alumni.unav.es (M.S.R.); fprosper@unav.es (F.P.); 3 Instituto de Investigación Sanitaria de Navarra (IdiSNA), 31008 Pamplona, Spain; aurbiola@unav.es (A.U.); cederra@unav.es (C.E.); mikelariz@unav.es (M.A.); codesolorzano@unav.es (C.O.-d.-S.); 4 Aragón Institute of Engineering Research (I3A), University of Zaragoza, 50018 Zaragoza, Spain; jgrasa@unizar.es (J.G.); bcalvo@unizar.es (B.C.); 5 Centro de Investigación Biomédica en Red en Bioingeniería, Biomateriales y Nanomedicina (CIBER-BBN), 28029 Madrid, Spain; 6 Imaging Platform, Foundation for Applied Medical Research (FIMA), University of Navarra (UNAV), 31008 Pamplona, Spain; 7 Haematology Department, Clínica Universidad de Navarra, 31008 Pamplona, Spain

**Keywords:** Achilles tendon, muscular degeneration, fatty infiltration, satellite cells, second-harmonic generation microscopy, muscle force

## Abstract

Achilles tendon rupture is a frequent injury with an increasing incidence. After clinical surgical repair, aimed at suturing the tendon stumps back into their original position, the repaired Achilles tendon is often plastically deformed and mechanically less strong than the pre-injured tissue, with muscle fatty degeneration contributing to function loss. Despite clinical outcomes, pre-clinical research has mainly focused on tendon structural repair, with a lack of knowledge regarding injury progression from tendon to muscle and its consequences on muscle degenerative/regenerative processes and function. Here, we characterize the morphological changes in the tendon, the myotendinous junction and muscle belly in a mouse model of Achilles tendon complete rupture, finding cellular and fatty infiltration, fibrotic tissue accumulation, muscle stem cell decline and collagen fiber disorganization. We use novel imaging technologies to accurately relate structural alterations in tendon fibers to pathological changes, which further explain the loss of muscle mechanical function after tendon rupture. The treatment of tendon injuries remains a challenge for orthopedics. Thus, the main goal of this study is to bridge the gap between clinicians’ knowledge and research to address the underlying pathophysiology of ruptured Achilles tendon and its consequences in the gastrocnemius. Such studies are necessary if current practices in regenerative medicine for Achilles tendon ruptures are to be improved.

## 1. Introduction

Tendon injuries are one of the most frequent musculoskeletal lesions, with four million new cases worldwide each year. They thus represent a significant burden for society and health assistance costs [1]. Despite its strength and thickness, the Achilles is one of the most frequently injured tendons. The incidence of Achilles tendon rupture is rising, probably due to the increase in the practice of sports [2,3,4]. Achilles tendon rupture is most common in middle age, affecting the general public participating intermittently in sporting activities but is also a frequent tendon injury among qualified athletes [5]. However, elderly and sedentary population may suffer discrete ruptures, which are usually missed and later become chronic, with a higher health-care cost (~EUR 6500) than treatments aimed to restore acute ruptures (~EUR 6000 and EUR 4500, operative and non-operative treatments, respectively) [6].

The Achilles tendon is the strongest and largest tendon in the body, designed to transmit traction forces from the calf muscles to the calcaneus (heel bone), resulting in supremely enduring tissue that enables foot movement during walking, running or jumping. It is able to withstand loads greater than 12 times body weight [7]. The Achilles tendon is characterized by a highly hierarchical organized structure, mainly composed of ordered type I collagen fascicles parallel to its long axis, that enables and regulates its function [8]. This perfect assemblage, from microfibrils to fascicles, provides the Achilles tendon with non-linear, viscoelastic and anisotropic mechanical properties to sustain the strength necessary to transmit loads from the gastrocnemius to the calcaneus [9]. Given its important role in ambulation, injury to the Achilles tendon can be debilitating not only because of the obvious loss of acute function, but also for its consequences on the gastrocnemius and soleus muscles [10,11,12,13,14,15]. After Achilles tendon rupture, the low cellularity and vascularity of the tendon tissue contribute to a slow and inefficient repair process, often with collagen organization disruption, resulting in mechanically, structurally and functionally inferior tissue [16]. Tendon ruptures resulting in lesions greater than 5 mm have limited healing capacity and result in loss of tissue function, meaning that surgery is the best option. Surgical treatment aims to restore the repaired tendon to its original length. However, although both ends of the ruptured tendon are debrided and correctly positioned together, the repaired tissue is often thickness and plastically deformed because of extensive scarring and adherence to overlying tissues and therefore not as mechanically strong as the pre-damaged tendon [17]. Current clinical treatments are still far from achieving complete restoration of the original tendon since they are still associated with short-term mechanical failure and long-term complications with the risk of secondary tendon ruptures [18].

Clinically, muscle atrophy and fatty infiltration related to Achilles tendon rupture have been observed in the gastrosoleus muscles, even 7 years after tissue repair, with muscle fatty degeneration being negatively associated with functional loss [19]. This situation is not restricted to complete ruptures of the Achilles tendon, as fatty degeneration has also been detected in diverse Achilles tendon abnormalities such as tendinopathies and partial ruptures [11]. Despite clinical findings suggesting the importance of muscle degeneration after Achilles tendon rupture, preclinical research in animal models does not include the effect of tendon injury in the muscle, but rather has focused on the investigation on tendon degeneration, repair and reconstruction [20,21,22]. Muscle fatty degeneration is well described in rotator cuff ruptures, both at the clinical and research levels [23]. Immediately after tendon rupture, damage to the rotator muscles occurs, leading to an inflammatory process that initiates muscle repair followed by muscle atrophy, fatty infiltration, retraction and fibrosis. Numerous investigations have described in detail the progression of rotator cuff injuries and the efficiency of regenerative approaches at the morphological and mechanical levels, supporting muscle fatty infiltration as being responsible for disease progression and unsuccessful outcomes in reparative surgeries [24]. Nevertheless, there is still relatively little knowledge regarding the consequences of Achilles tendon rupture/recovery [25] and considerably less information related to the Achilles tendon-gastrocnemius unit, which is crucial in fully understanding tendon mechanics. Through an improved understanding of these processes, enhanced techniques for Achilles tendon repair and regeneration may be developed.

Here, we investigated the natural course of Achilles tendon complete rupture in a mouse injury model after complete resection, without any postoperative immobilization. We describe the main morphological changes occurring in the tendon itself and in the gastrocnemius, highlighting injury progression from tendon to muscle and the consequences on muscle mechanical properties. Treatment of tendon injuries is of great concern in orthopedics, and this study opens up new opportunities for innovative investigation by offering an in-depth analysis of the underlying pathophysiology of ruptured Achilles tendon and its repercussion in the gastrocnemius.

## 2. Materials and Methods

### 2.1. Model of Injury

Male and female wild-type (WT, C57BL/6J) mice were purchased from ENVIGO and maintained in our animal housing facilities, according to current legislation. All animal procedures performed in this study were subjected to the European and Spanish legal regulations (protocol number 095/19, approved on 11 December 2019).

Animals underwent surgery at the age of 11–12 weeks, with a median weight of 21.8 gr ± 0.4 (22.7 gr ± 1.3 for males and 20 gr ± 0.6 for females). The right lower limb was operated on by performing a complete tenotomy for the Achilles tendons. Flexor hallucis longus tendons were also resected to avoid their hypertrophic compensatory response after acute Achilles injury [13]. The contralateral limb was used as internal control. Surgical procedures were carried out under 2% isoflurane inhalation anesthesia with aseptic conditions and optical magnification (SZ61, Olympus). The right lower limbs of the mice were shaved and disinfected with chlorhexidine. With the animal in a prone position, a longitudinal incision was made lateral to the distal part of the triceps surae down to the calcaneus, and a careful dissection exposed the Achilles and the flexor hallucis longus tendons. A complete tenotomy was performed in both tendons, 2 mm proximal to their distal insertion, followed by a 2 mm tendon resection, including the paratenon, marking the proximal stump with suture for its subsequent location (Prolene 7/0, Johnson & Johnson, New Brunswick, NJ, USA). The animals were kept in cages with environmental enrichment, with access to food and water ad libitum. Weekly, they were examined and weighed to check their correct development. Antibiotic prophylaxis (enrofloxacin 10 mg/kg) and analgesic medication (Fentanest 300 μg/kg, meloxicam 0.04 mg/10 g) were administered to all specimens before and after surgery.

The animals were sacrificed by cervical dislocation at 2, 14, 28 and 60 days after tenotomy. The Achilles tendon and the gastrocnemius were isolated for their histological and mechanical characterization. The tissues from healthy contralateral limbs were used as controls.

### 2.2. Immunostaining

Gastrocnemius were frozen in isopentane cooled in liquid nitrogen, and serial 9-μm cryosections were collected at 100-μm intervals through the entire muscle. Achilles tendons were immersed into 15% sucrose/PBS for 30 min and then into 30% of sucrose/PBS overnight at 4 °C. Finally, tendons were embedded in O.C.T. compound (Tissue-Tek), frozen on dry ice and serially cryosectioned in 20-μm sections. Tissue sections were fixed in 10% formalin for 20 min and rinsed in PBS. When required, sections were permeabilized with 0.1% Triton X-100/PBS, citrate buffer or treated with pepsin/colagenase, and blocked.

The following primary antibodies were applied (4 °C, overnight): mouse anti-Pax7 (Developmental Studies Hybridoma Bank, DSHB), rat anti-Ki67 (eBioscience, San Diego, CA, USA; SolA15 clone; Ref.: 14569882), anti-CD45 (Biolegend, San Diego, CA, USA; 30-F11 clone; Ref.: 103102), rabbit anti-collagen I (Abcam; Ref.: ab34710), anti-perilipin (Cell Signaling, Danvers, MA, USA; D1D8; Ref.: 9349S) and anti-laminin (Sigma, St. Louis, MO, USA; Ref.: L9393). Primary antibodies were visualized with fluorochrome-conjugated secondary antibodies (Molecular Probes) before mounting in Faramount fluorescent mounting medium containing 4,6-diamidino-2-phenylindole (DAPI; 100 ng/mL; Molecular Probes).

For multiphoton analyses, the calcaneus-Achilles-gastrocnemius units were collected, fixed in 4% paraformaldehyde for 24 h and decalcified for 72 h at room temperature. Then, bone-tendon-muscle units were embedded in agarose and longitudinal sectioned in 300- to 500-μm depth intervals. Immunostainings of tissue sections for dystrophin (Abcam) and perilipin were performed as indicated above but increasing time incubations (24 h) at room temperature.

Sirius Red and H&E staining were performed as previously described elsewhere [26].

### 2.3. Multiphoton Image Acquisition

Images were collected using a Zeiss LSM 880 (Carl Zeiss, Jena, Germany) equipped with a two-photon femtosecond pulsed laser (MaiTai DeepSee, Spectra-Physics, Newport, RI, USA), tuned to a central wavelength of 790 nm using a 25×/0.8 objective (LD LCI Plan-Apochromat 25×/0.8, Carl Zeiss, Jena, Germany). Tile and z-stack scans from 300- to 500-µm sections were acquired in non-descanned mode after spectral separation and emission filtering using 380- to 430-nm and 465- to 515-nm BP filters for SHG and perilipin or dystrophin signals, respectively.

### 2.4. Digital Image Acquisition and Quantification

Immunostained muscle tissue sections were imaged using a Zeiss Axiophot epifluorescence microscope. The digital images were processed using AxioVision and ImageJ software. When needed, images were composed and edited, and modifications were applied to the whole images using Photoshop CS6 (Adobe, 2015.1 version, Mountain View, CA, USA). In muscle tissue sections, positive staining for perilipin and CD45 was expressed as the percentage of the stained area divided by the total area of muscle. Pax7^+^Ki67^+^ and Pax7^+^Ki67^−^ satellite cells were counted in the whole muscle section and related to the area of the muscle.

Picrosirius red and H&E stained sections were scanned in an automated pathology imaging system. Tissue samples were analyzed under polarized light.

Immunostainings were performed in four different tissue sections from the same specimen for each time point analyzed, and then average data were calculated for each sample. Three (2 dpi) and six (14, 28 and 60 dpi) mice were analyzed.

For quantification of fatty infiltration by multiphoton microscopy 60 days after tenotomy, four different images from the same healthy or damaged leg were analyzed, including three independent mice for each condition. The analysis of the 3D stacks was carried out using a plugin developed for Fiji/ImageJ, an open-source Java-based image processing software (Image J2 version, University of Wisconsin, Madison, WI, USA) [27], and designed by the Imaging Platform at the Center for Applied Medical Research (CIMA). SGH and perilipin channels were first split. The SGH signal was thresholded, and the resulting binary mask was denoised to obtain the tendon segmentation. Perilipin channel was also thresholded and denoised, and morphological post-processing was applied to eliminate signals that corresponded to muscle autofluorescence. The resulting binary mask was further corrected manually to eliminate remaining artifacts and obtain a reliable adipocyte segmentation. Tendon and fat volumes were quantified from their corresponding segmentation masks, and the ratio of fat with respect to tendon volume was calculated as a normalized measure of fat content.

### 2.5. Morphometric Analysis

Morphometry was used to determine the number of fibers and the cross-sectional area of each muscle fiber 60 days after tenotomy. For this, laminin immunofluorescence of whole muscle sections was analyzed, recording the CSA for all muscle fibers or for perinuclear fibers and centronuclear regenerated fibers, separately. Necrotic fibers, identified by the presence of multiple nuclei and no defined laminin immunostaining (Figure S1), were discarded. The CSA was measured in µm^2^, and the number of fibers was calculated as the number of fibers per µm^2^ of muscle. Analyses were carried out in two different muscle tissue sections from the same specimen. Four independent mice were assessed.

### 2.6. Muscular Mechanical Characterization

The distal muscle end was fixed inside an organ bath (20 × 20 × 20 cm), while the proximal end was fixed to the actuator of an electromechanical Instron Microtester 5248 (Illinois Tool Works, Inc., Glenview, IL, USA) with a 5 N full scale load cell [28]. The temperature of the Ringer’s bath solution was maintained at 27 °C and saturated with carbogen gas. The muscles were stimulated using a pair of platinum plate electrodes connected to a CIBERTEC CS-20 electrical signal generator [28].

A series of electrical pulses (1 ms and 60 V) were applied to all the muscle samples and the isometric force was registered for different muscle lengths. The length at which the muscle twitch was maximum was considered as the optimal length of each muscle sample. After optimal length determination, the control group was used to determine the optimal stimulation parameters (voltage and frequency) to obtain the maximum isometric force. Thereby, electrical pulses from 40 to 100 V were applied to the muscle for 1 ms each. An interval of two minutes was left between stimuli. At the end, the sample rested for five minutes. Then, trains of pulses at increasing frequencies (from 20 to 100 Hz) were applied. Finally, 100 V and 90 Hz were chosen as the optimal parameters of muscle stimulation, and they were used to stimulate the damaged muscles.

To register muscle active forces, all samples were stimulated with 3 trains of pulses at 90 Hz, 100 V and 500 ms. These active tests allowed us to monitor the active behavior of the muscle, or capacity to produce force, over the time.

Quantification of muscle isometric force was carried out in six (14 dpi) and four (28 and 60 dpi) different biological replicates.

### 2.7. Statistical Analysis

All statistical analyses were performed using SPSS 26.0 (SPSS, Inc., Armonk, NY, USA). The Shapiro-Wilk test was used to assess normal distribution. Variables were analyzed with the Mann–Whitney U test or Student *t* tests. All experiments were performed using at least three independent experiments per each condition. Data are expressed as means ± SEM. *p* values < 0.05 were considered to be statistically significant.

## 3. Results

### 3.1. Morphological Changes in the Achilles Tendon after Complete Rupture

**Figure 1:**
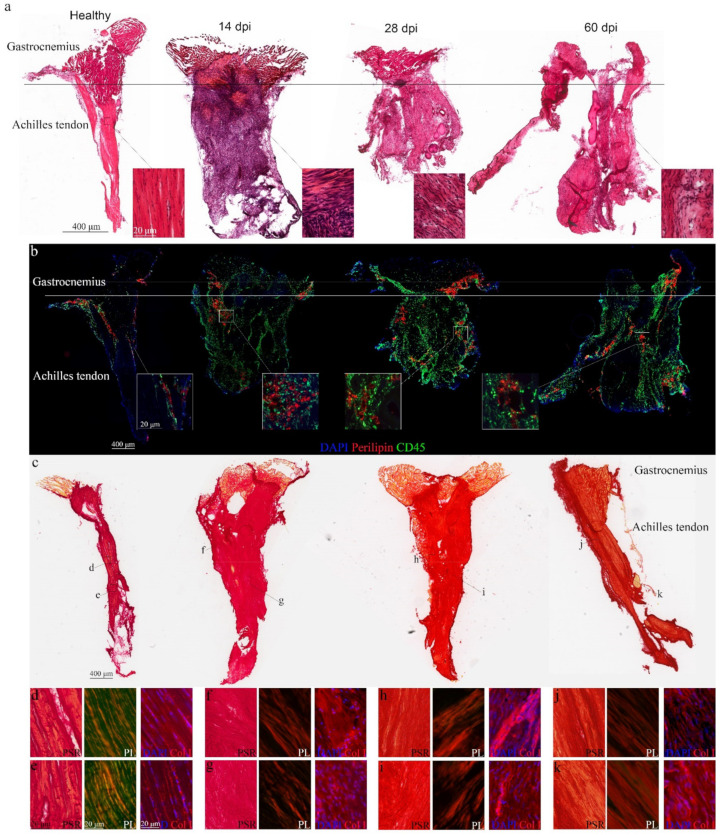
Characterization of damage in Achilles tendon after complete rupture. (**a**) Representative tissue sections of healthy and injured Achilles tendons stained for H&E; image scale bar 400 μm; insert scale bar 20 μm; (**b**) with inserts highlighting tissue microstructure in healthy and injured Achilles tendons and representative images of tendons immunostained for CD45/perilipin; image scale bar 400 μm; insert scale bar 20 μm; (**c**) Picrosirius red staining of tissue cryosections; image scale bar 400 μm; and (**d**–**k**) inserts pointing to areas visualizing collagen fiber arrangement under normal or polarized light and after collagen I immunostaining; image scale bar 20 μm. DAPI was used to identify all nuclei. dpi, days post-injury; PSR, Picrosirius red; Col I, collagen type I.

We reproduced Achilles tendon rupture by resecting 2 mm of the tissue in adult wild-type mice. After 14, 28 and 60 days, animals were sacrificed and tendons longitudinally cryo-sectioned. Healthy tendons maintained continuous and aligned fibers, and low cellularity, with cells typically arranged in longitudinal rows, in parallel to the long axis of the whole tendon itself (Figure 1a). This pattern changed 14 days after tenotomy, with the Achilles tendons completely losing their organized structure with total disruption of the tendon fibers and high presence of inflammatory cells invading the tissue (Figure 1a). This deleterious situation persisted at 28 and 60 days (Figure 1a). Co-immunostaining of tendon tissue sections for CD45 and perilipin in addition to confirming the accumulation of infiltrating cells within the injured tendon, detected fatty infiltration not only in the tendon itself but also in the myotendinous junction (Figure 1b). Collagen fiber arrangement was analyzed by Picrosirius red and collagen I staining, confirming that the organized collagen pattern along the whole healthy tendon (Figure 1c–e) changed after injury (Figure 1c,f–k). Collagen fibers were organized in a mesh-like arrangement 14 and 28 days after rupture. A few areas, those closer to the tendon-muscle connection, presented a better collagen bundle arrangement but with increased cellularity (Figure 1f–i). Two months after injury, collagen organization increased, with some collagen bundles regularly ordered with the same orientation as the tendon and cells beginning to align within the axis of the bundles (Figure 1j,k).

Our findings verify Achilles tendon microstructural changes after complete rupture, with fatty infiltration as a new marker of damage progression.

### 3.2. Pathological Progression in the Gastrocnemius after Achilles Tendon Complete Rupture

**Figure 2:**
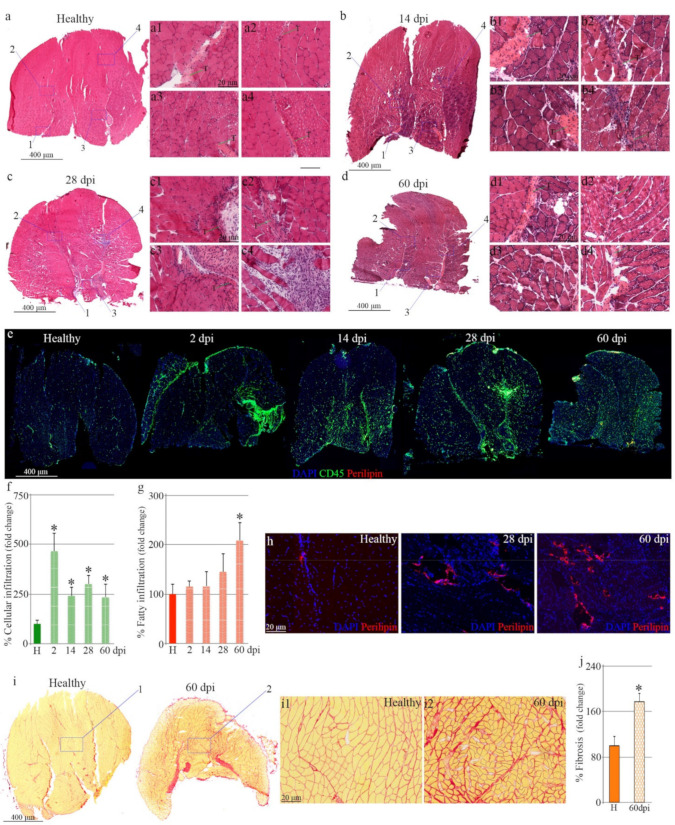
Characterization of damage in the gastrocnemius after complete tendon rupture. (**a**) Representative muscle tissue sections of healthy and injured hind limbs stained for H&E with (**a1**–**d4**; T) inserts highlighting muscle microstructure in areas closer to tendon insertion. Representative images of skeletal muscles immunostained for (**e**) CD45/perilipin and (**h**) higher magnification of specific areas positive for perilipin. Graphs show the percentage of (**f**) immune cells and(**g**) fatty infiltration localized in muscles of control and injured tendons. (**i**) Picrosirius red staining of muscle cryosections and (**i1**,**2**) inserts pointing to areas of higher magnification. (**j**) Graph showing the percentage of connective tissue accumulation in muscles from healthy or injured limbs. DAPI was used to identify all nuclei. Bars in f, g and j show the mean ± SEM of at least three independent experiments (*n* = 3, 2 dpi; *n* = 6, 14 dpi; *n* = 6, 28 dpi; *n* = 6, 60 dpi). * designates significance between healthy and injured experimental groups. dpi, days post-injury; H, healthy.

To relate tendon changes after injury to microstructural alterations in the connected skeletal muscle, the gastrocnemius was isolated from mice after tenotomy and compared to muscles extracted from controls (Figure 2a). Transversal sections from healthy muscles showed an organized muscle structure composed of thousands of fibers with most myonuclei localized in the periphery and rare nuclei confined to a centralized position, indicative of activated muscle stem cells (Figure 2(a1–4)). In contrast, two days after tenotomy, muscle composition changed, with infiltrating cells invading the muscle (Figure S2a–e, black arrowheads) and myofibers undergoing necrosis (Figure S2d,e, white arrowheads). Muscles isolated 14 days after tendon rupture had some areas with necrotic myofibers, regenerating muscle fibers and infiltrating cells localized in the areas neighboring the tendon (Figure 2(b1–4). These muscle changes were more evident 28 days after tendon injury, with the detection of adipocytes within the muscles (Figure 2(c1–4)). By two months, increased fatty infiltration, connective tissue accumulation and cell infiltration characterized the muscle tissue sections from ruptured tendons (Figure 2(d1–4)). Most muscle alterations were visualized in sections closer to the tendon insertion site, showing damage progression from the ruptured tendon to the myotendon unit and muscle belly. Furthermore, we also observed Achilles tendon structure impairment along its insertion into the muscle, compared to healthy tendons (Figure 2a–d).

The muscle deterioration phenotype after tenotomy was confirmed by immunostaining, finding a 5-fold increase of CD45^+^ infiltrating cells two days after damage. This increase declined later but was still significantly higher compared to healthy muscles (Figure 2e,f). Fatty infiltration was identified by day 28 and further increased one month later (Figure 2e,g,h) when the abundance of connective tissue was significantly evident in injured muscles as compared to muscles from control legs (Figure 2i,j).

Taken together, our data confirm gastrocnemius injury progression after Achilles tendon complete rupture.

### 3.3. Loss of Self-Renewed Satellite Cells after Achilles Tendon Rupture

**Figure 3:**
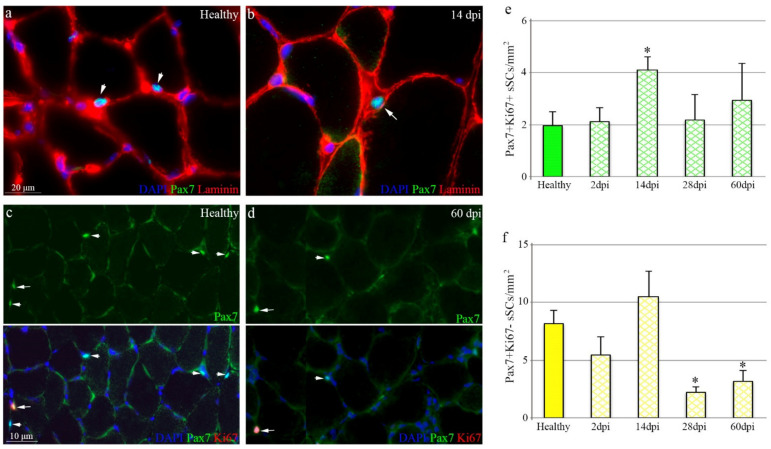
Satellite cell compartment is compromised after chronic tendon rupture. Representative muscle tissue sections from healthy and injured tendons immunostained for (**a**,**b**) Pax7/laminin and (**d**,**e**) Pax7/Ki67. Arrows and arrowheads indicate quiescent and activated/proliferating Pax7^+^ satellite cells, respectively. DAPI was used to identify all nuclei. Graphs show the number of total (**c**) Pax7^+^Ki67^+^ and (**f**) Pax7^+^Ki67^−^ satellite cells per µm^2^ in the muscles of experimental groups. Values are presented as the average of at least three independent experiments (mean ± SEM), where * designates significance (*p* < 0.05) between muscles from healthy and injured tendons. (*n* = 3, 2 dpi; *n* = 6, 14 dpi; *n* = 6, 28 dpi; *n* = 6, 60 dpi). dpi, days post injury.

Muscle repair response depends on the satellite cells, the adult stem cells of the skeletal muscle, which have the capacity to provide more satellite cells that differentiate into muscle, while replenishing the stem cell compartment [29]. Healthy muscles had dormant Pax7^+^ satellite cells under the basal lamina of the fibers (Figure 3a, arrowheads). After tendon rupture, satellite cells became activated, occupying the external side of the myofibers (Figure 3b, arrows). By immunostaining, we were able to monitor the presence of quiescent (Ki67^−^) and activated/proliferating (Ki67^+^) Pax7^+^ satellite cells in the gastrocnemius after damage (Figure 3c–f). The number of Pax7^+^Ki67^+^ satellite cells, whose function is to repair muscle, significantly increased 14 days after Achilles tendon rupture but then dropped to numbers of healthy muscles (Figure 3c–e, arrows). Interestingly, the self-renewed Pax7^+^Ki67^−^ satellite cells, destined to replenish the stem cell pool, significantly decreased from day 28 after tenotomy (Figure 3c,d,f, arrowheads), as compared to healthy muscles.

Overall, these findings suggest impairment of skeletal muscle regenerative capacity after tendon injury, with a significantly deficient muscle stem cell supply.

### 3.4. Muscular Function Decline after Achilles Tendon Rupture

**Figure 4:**
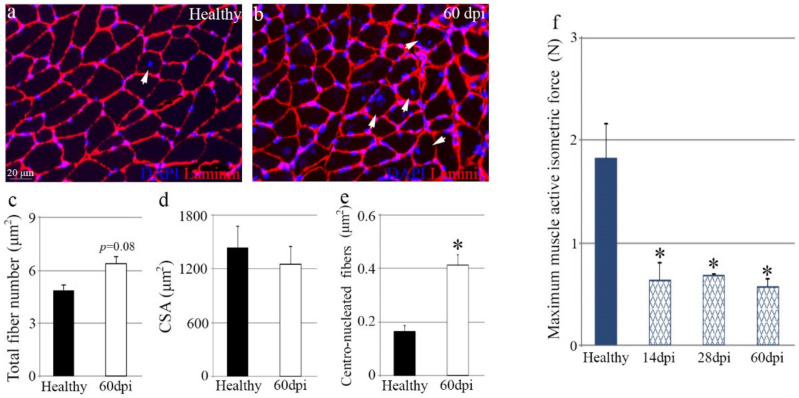
Muscle function declines after chronic tendon rupture. (**a**,**b**) Representative images of tissue sections of muscles from healthy and injured tendons immunostained for laminin. Arrowheads identify regenerating myofibers with centralized nuclei. DAPI was used to identify all nuclei. Graphs showing the (**c**) total number of fibers in the whole muscle tissue section, (**d**) average CSA and (**e**) the number of myofibers with centralized nuclei of healthy and injured muscles (*n* = 4). (**f**) Quantification of muscle isometric force was carried out in six (14 dpi) and four (28 and 60 dpi) biological replicates. Bars represent the mean ± SEM, where * designates significance between muscles from healthy tendons and muscles from injured tendons. dpi, days post-injury; N, Newton.

Next, we analyzed muscle function two months after tendon rupture by performing a morphometric analysis of muscle tissue sections immunostained for laminin (Figure 4a,b). Damaged muscles showed a slight increase in the total number of fibers compared to healthy muscles (Figure 4c). However, the average cross-sectional area of myofibers in injured muscles did not differ from that of contralateral undamaged muscles (Figure 4d). Interestingly, tendon rupture led to increased numbers of myofibers with centralized nuclei (Figure 4e), suggesting that the muscle repair response was still activated 60 days after injury.

In order to accurately measure muscle function in a physiological condition, muscles were isolated and immediately subjected to local stimuli to assess isometric forces. Muscles connected to ruptured tendons exhibited a notable loss of mechanical function after injury, with a 65% decline in muscle force 14 days after tendon rupture (Figure 4f). This maximal force decrease was maintained for 60 days (Figure 4f).

### 3.5. Tendon-Skeletal Muscle Structure after Achilles Tendon Complete Rupture

**Figure 5:**
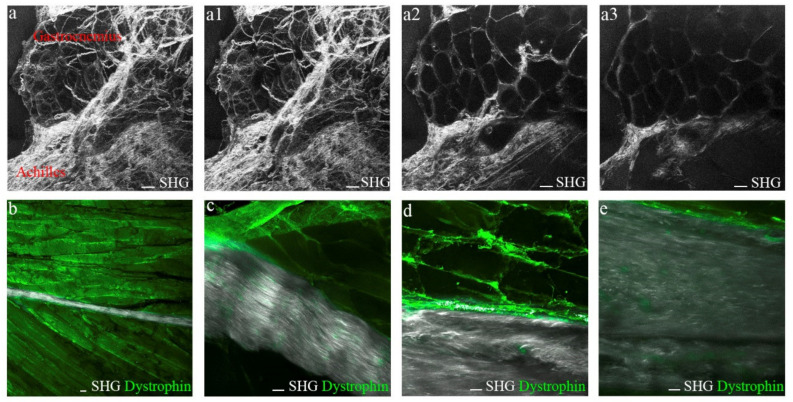
SHG signals from collagen fibers of muscle and tendon origin. (**a**–**a3**) Representative multiphoton images of the Achilles tendon-gastrocnemius unit from healthy mice. (**a**) Maximal Z-stack projection image and (**a1**–**3**) representative images from different Zs. Scale bar 100 µm. Representative SHG collagen signals combined with dystrophin immunostaining of (**b**,**c**) healthy and (**d**,**e**) injured Achilles tendons. Scale bar 20 µm. SHG, second-harmonic generation.

Second-harmonic generation (SHG) is a non-linear microscopy modality highly sensitive to the collagen fibril/fiber structure [30]. Thus, we used SHG to obtain structural information on the assembly of collagen fibers in the Achilles tendon after rupture, in the tendon itself and at its insertion into the gastrocnemius. First, we analyzed the intact animal’s hind limbs using two-photon excitation, confirming SHG signaling from collagen fibers within the tendon and the skeletal muscle (Figure 5a). Then, the hind extremities were sectioned from the heel bone to the distal femur, preserving the integrity of the Achilles tendons and gastrocnemius. By combining SHG and classical fluorescence, we accurately distinguished between tendon and muscle tissues after dystrophin immunostaining, a specific protein expressed by the skeletal muscle [31], both in healthy (Figure 5b,c) and injured tissues (Figure 5d,e).

**Figure 6:**
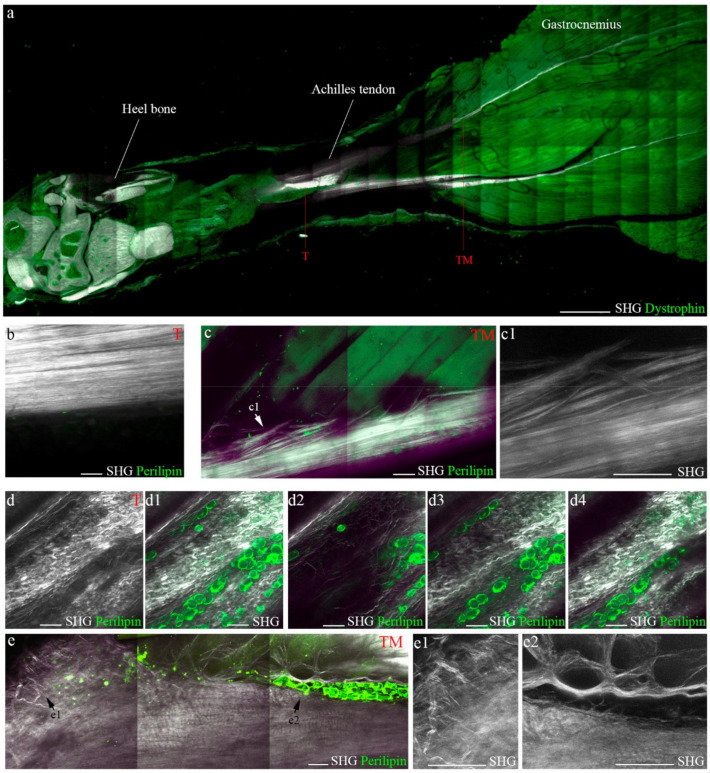
SHG signals monitor damage after Achilles tendon complete rupture. (**a**) Representative multiphoton image of the heel bone-Achilles-gastrocnemius unit combined with dystrophin immunostaining, highlighting areas in the tendon itself (T) and at the tendon-muscle connection (TM). Scale bar 1 mm. Representative images of SHG-perilipin immunostaining in (**b**–**c1**) healthy and (**d**–**e2**) injured tissues. (**c1**,**d1**–**d4**,**e1**,**e2**) are high magnification images of (**c**–**e**) pictures. Scale bar 50 µm. SHG, second-harmonic generation.

We analyzed the structure of healthy and ruptured tendons 60 days after lesion in different areas, from neighboring sites of its insertion into the calcaneus (heel bone) to its origin at the lower margin of the gastrocnemius belly (Figure 6a). In the healthy tendon itself, collagen fibers had a continuous rippled aligned appearance (Figure 6b), and this tidy orientation and alignment continued at the myotendinous junction in the muscle belly (Figure 6c). In contrast, collagen fibers completely lost their wavy ordered aspect in areas close to the rupture site. Adipocytes, which generate third-harmonic generation (THG) rather than SHG signals [32], were detected invading disorganized collagen fibers by perilipin-specific immunofluorescence (Figure 6d). In the tendon-muscle unit, adipocytes accumulated in the tissues, with collagen fiber anomaly aligned in the tendons and muscles (Figure 6e). Quantification of perilipin expression from SHG imaging data confirmed a significant fatty infiltration increase in tissues 60 days after tenotomy, compared to healthy sections (Figure S3).

Taken together, our findings demonstrate the possibility of monitoring fatty infiltration and collagen fiber arrangement after Achilles tendon complete rupture using multiphoton technology combined with classical fluorescence.

## 4. Discussion

The incidence of Achilles tendon rupture has increased in recent decades. These injuries frequently occur in the third or fourth decade of life in non-top-level training individuals and are frequent in elite athletes [5]. However, treatment of ruptured tendons currently remains a challenge, with repair often resulting in inferior quality tissue and long-term complications and morbidity for patients.

Recent investigation of tissue and cells from tendons such as the rotator cuff has demonstrated the importance of inflammation and fatty infiltration in the development of tendon disease, underscoring the consequent injury to muscle. Immediately after tendon rupture, damage to rotator muscles occurs, with the recruitment of inflammatory cells that initiate a muscle repair response. If injury is not promptly resolved, muscle atrophy, fatty infiltration, retraction and fibrosis aggravate tendon injury, which in turn further impair and endanger surgical repair of rotator cuff tears [23]. These mechanisms remain poorly understood when the rupture occurs in tendons such as the Achilles, and less information is available regarding the consequences of injury at its corresponding muscle, the gastrocnemius [33]. Clinically, muscle atrophy and fatty infiltration have been detected in the gastrocnemius even years after Achilles tendon repair, and these have a negative correlation with functional outcomes [19]. However, although some clinical studies have described muscle degeneration after Achilles tendon rupture [11,13,14], almost all analyses focus on repairing the tendon itself, without considering the accompanying muscle degeneration [2,3,4]. Importantly then, our study highlights the relevance of describing in detail the damage to the tendon and muscle, not separately but as a unit after Achilles rupture. Indeed, we found an injury/repair pattern characterized by an inflammatory response after damage, followed by fibrosis and fatty infiltration in the tendon itself, myotendinous unit and muscle belly, demonstrating tissue changes from tendon to muscle. As a consequence of tendon tension loss, muscle force significantly decreased.

Fatty muscular degeneration is being thoroughly investigated in clinical and pre-clinical models of muscular diseases [34]. With regard to tendon injuries, fatty infiltration in muscle is responsible for poor outcomes after rotator cuff tears [23]. The origin of muscle fatty degeneration relies on the excessive or persistent activation of the healing response after injury. In this acute scenario, two stem cell populations within the skeletal muscle compete, the satellite cells, with the capacity to restore muscle, and the fibro-adipogenic precursor cells (FAPs), with the potential to differentiate into fibrotic and adipocyte cells [35,36]. Although FAPs are essential to activate dormant satellite cells and initiate the muscle repair response, excessive signaling of FAPs through inflammatory cells exceeds the regenerative capacity of the satellite cells, and consequently, muscle is replaced by fibrotic and fatty tissue [35,36,37,38,39]. Our results are in agreement with this pattern, finding a persistent inflammatory response in the gastrocnemius after tendon rupture, with a significant decline of quiescent satellite cells and accumulation of connective tissue and fatty infiltration. Furthermore, we have detected an acute inflammatory response and fatty infiltration in the Achilles tendon itself and in the myotendon unit after rupture. Thus, in addition to monitoring the presence of FAPs during the muscle repair response, in future studies it will be interesting to address the existence of these mesenchymal progenitors in the tendon itself, as has been recently suggested by others [40], as well as their relation to tenocytes and tendon stem/progenitor cells.

Collagen is the major component of the extracellular matrix of the tendon, which determines its mechanical properties, including stiffness and flexibility. Alterations of the spatial structure of the collagen are responsible for tendon ruptures that can further cause secondary episodes of rupture in surgically or conservatively repaired tendons [41,42]. Therefore, accurate characterization of collagen structures may be of great interest to understand the properties of healthy and diseased tendons and enable the establishment of innovative diagnostic tools and therapeutic strategies. The classical technique used to visualize fibrillar collagen structure is based on polarized transmitted light microscopy after staining histological tissues with Picrosirius red [43]. However, recent studies have cast doubt on the use of this method to study collagen network in different tissues, question the possibility of carrying out a quantitative morphometric analysis by differentiating collagen bundles and collagen types, or query whether it only reflects fiber thickness and packing [44]. Other imaging techniques, such as transmission electron microscopy, atomic force microscopy, reflectance confocal microscopy and optical coherent tomography, are available to assess collagen fiber arrangement at various spatial resolutions, providing 3D images of unstained ex vivo and in vivo samples [45,46,47,48]. However, the second-harmonic generation (SHG) multiphoton technique is currently the “gold standard” method for in situ studies of unstained collagen tissues. The hierarchical structure of collagen fibers contributes to their intrinsic ability to produce strong non-linear emission of SHG signals [49], thus providing 3D images of the morphology and spatial distribution of collagen fibers with unrivalled specificity and contrast [50,51,52]. Here, we have demonstrated that SHG microscopy enables the visualization of collagen fiber arrangement in the Achilles tendon under healthy and pathological conditions, revealing changes in its wavy ordered pattern and organization in the tendon itself, myotendon unit and muscle belly. Furthermore, we have combined multiphoton technology and fluorescence to track with greater accuracy the changes occurring at the tendon-muscle connection, as previously reported [53], to easily monitor fatty infiltration in tendons and muscles, while analyzing its effect on fiber arrangement. Furthermore, this technology may permit further investigation of the bone–tendon–muscle connection, as we have observed collagen SHG signals from the heel bones. However, development of quantitative methods and a comprehensive strategy possibly using advanced machine learning tools will be necessary to assess the disease state after tendon injury/recovery.

Our study has some limitations, such as the use of contralateral undamaged limbs as tissue controls instead of using a sham group, which can underestimate our findings. Operated animals can overuse their un-injured legs and develop compensatory mechanisms, which may conceal bigger differences between control and damaged tissues. Furthermore, viscoelastic and non-linear mechanical properties of the Achilles tendons should be examined in future studies, since disorganization of the collagen hierarchical structure is the major responsible factor for tendon function loss.

## Data Availability

Data can be obtained from the corresponding authors on request.

## Supplementary Materials

The following are available online at https://www.mdpi.com/article/10.3390/biomedicines10010019/s1: Figure S1. Skeletal muscle tissue sections immunostained for laminin with arrowheads identifying necrotic fibers. DAPI was used to distinguish all nuclei. Figure S2. Representative muscle sections of healthy and injured hind limbs two days after tenotomy. Black arrowheads in a-e identify infiltrating cells, while white arrowheads in d and e point necrotic fibers. Figure S3. Quantification of fatty infiltration in healthy and injured mice measured from SHG/Perilipin images. Values represent the mean ± SEM from three independent mice, where * designates significance between experimental groups. dpi, days post injury.
