# Supplementary material for: Muscular and Tendon Degeneration after Achilles Rupture: New Insights into Future Repair Strategies
Source: Biomedicines. 2021 Dec 23;10(1):19. doi: 10.3390/biomedicines10010019 (PMC8773411; doi:10.3390/biomedicines10010019)
Supplement: Supplementary file 1 [file biomedicines-10-00019-s001.zip › biomedicines-1471528-supplementary.pdf]

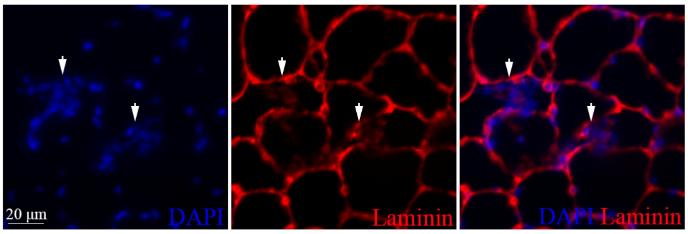

Figure S1. Skeletal muscle tissue sections immunostained for laminin with arrowheads identifying necrotic fibers. DAPI was used to distinguish all nuclei.

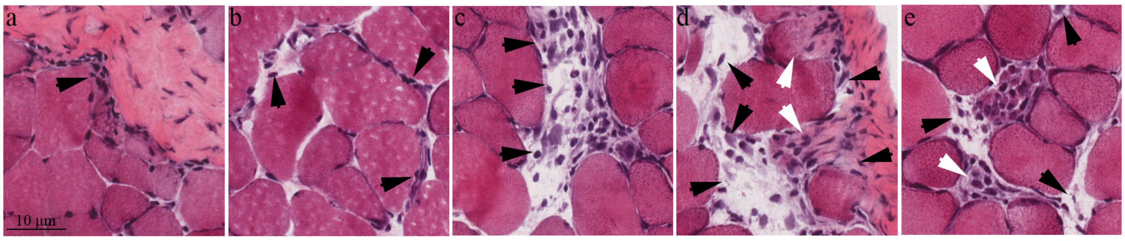

Figure S2. Representative muscle sections of healthy and injured hind limbs two days after tenotomy. Black arrowheads in a-e identify infiltrating cells, while white arrowheads in d and e point necrotic fibers.

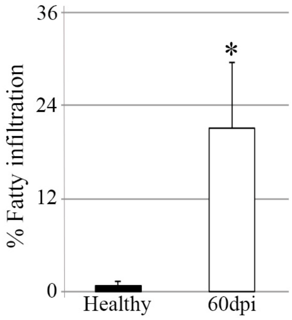

Figure S3. Quantification of fatty infiltration in healthy and injured mice measured from SHG/Perilipin images. Values represent the mean ± SEM from three independent mice, where * designates significance between experimental groups. dpi, days post injury.
